# What can we learn about emotion by studying psychopathy?

**DOI:** 10.3389/fnhum.2013.00181

**Published:** 2013-05-10

**Authors:** Abigail A. Marsh

**Affiliations:** Department of Psychology, Georgetown UniversityWashington, DC, USA

**Keywords:** psychopathy, emotion, amygdala, empathy, fear

## Abstract

Psychopathy is a developmental disorder associated with core affective traits, such as low empathy, guilt, and remorse, and with antisocial and aggressive behaviors. Recent neurocognitive and neuroimaging studies of psychopathy in both institutionalized and community samples have begun to illuminate the basis of this condition, in particular the ways that psychopathy affects the experience and recognition of fear. In this review, I will consider how understanding emotional processes in psychopathy can shed light on the three questions central to the study of emotion: (1) Are emotions discrete, qualitatively distinct phenomena, or quantitatively varying phenomena best described in terms of dimensions like arousal and valence? (2) What are the brain structures involved in generating specific emotions like fear, if any? And (3) how do our own experiences of emotion pertain to our perceptions of and responses to others' emotion? I conclude that insights afforded by the study of psychopathy may provide better understanding of not only fundamental social phenomena like empathy and aggression, but of the basic emotional processes that motivate these behaviors.

Emotion is the major driver of all human and animal behavior, including social behavior—it is emotion that literally moves us to seek or escape positive and negative consequences (LeDoux, 2012). Many unanswered questions remain about the nature of human emotion and are the topic of vibrant ongoing debates: are different emotions qualitatively distinct, emerging from separable neurobiological processes, or can emotions be more accurately described dimensionally in terms of arousal and valence (Russell and Barrett, 1999; Barrett et al., 2007; Izard, 2007; Panksepp, 2007; LeDoux, 2012)? If distinct neurobiological events contribute to the generation of different emotions, which brain structures are most relevant to the emergence of these emotions (Panksepp, 2007; Vytal and Hamann, 2010; Lindquist et al., 2012)? And finally, how do emotions we experience pertain to our perceptions of and responses to emotions in others (Zahavi, 2008; Heberlein and Atkinson, 2009)?

Answering these questions about human emotion presents a variety of challenges. Unlike the study of some other human cognitive processes, the study of emotion benefits from the now widely accepted fact that humans and non-human animals share many emotional processes, enabling more, and more diverse study paradigms on emotion (Panksepp, 2007; Panksepp and Lahvis, 2011; LeDoux, 2012). A benefit of studying non-human animals is that they enable critical experimental manipulations to be performed, such as environmental manipulations that cause intense, ecologically valid experiences like fear, and manipulations of subcortical brain structures involved in emotion, such as permanent or temporary lesions or genetic manipulations. Gray and McNaughton argue that such techniques are essential for drawing causal inferences about some emotional processes (Gray and McNaughton, 2000). However, animals can provide little information relevant other critical features of emotion, such as information about subjective experiences. Research in humans can target subjective experience, but, conversely, many critical experimental manipulations of emotion are not feasible or ethical to perform in humans, such as intense, ecologically valid environmental manipulations or lesions to subcortical structures.

One means of circumventing this conundrum is to conduct research in individuals affected by pathologies that provide “natural experiments” in which emotional processes are altered, enabling identification of the downstream effects. One example is the use of case studies of individuals with lesions to specific brain regions as a result of disease, injury, or surgical intervention, such as the orbitofrontal cortex (Hornak et al., 2004), insula (Phillips et al., 1997), or amygdala (Feinstein et al., 2011). Such cases can yield rich and detailed evidence about the emotional processes subserved by the damaged region. The downside is that individuals in whom lesions are neuroanatomically specific enough to yield meaningful evidence are rare. Thus, few researchers have access to these patients, and the possibility persists that certain response patterns result from patient-specific idiosyncrasies unrelated to the lesion. In addition, most brain lesions occur in late adolescence or adulthood, precluding an understanding of the developmental consequences of lesions to structures like the amygdala, damage to which may result in distinct behavioral outcomes in adulthood relative to infancy (Amaral, 2003).

An alternative to lesion-based case studies is the study of populations of patients affected by psychopathologies known to affect specific neurocognitive systems. Psychopathy, a cluster of behavior tendencies and personality traits associated with callousness and antisocial behavior, is one such form of psychopathology (Hare, 1993; Blair et al., 2006; Skeem et al., 2011). Evidence is accumulating to suggest impairments in the systems and processes supporting fear responding in psychopathy, leaving other systems largely intact (Lilienfeld et al., 2012; Patrick et al., 2012; Rothemund et al., 2012). Psychopathy may therefore be a useful empirical tool for understanding the nature of fear and perhaps emotion more broadly.

In this review, I will consider how understanding psychopathy can shed light on the three questions outline above: (1) Are emotions discrete, qualitatively distinct phenomena or quantitatively varying phenomena best described in terms of dimensions like arousal and valence? (2) What are the brain structures involved in generating specific emotions like fear, if any? And (3) how do our own experiences of emotion pertain to our perceptions of and responses to others' emotion?

## Psychopathy

Psychopathy is a disorder that is generally viewed as the confluence of core personality characteristics plus antisocial behavioral tendencies, and which, in its extreme form, affects 1–2% of the general population and as many as 50% of violent offenders (Hare, 1993; Rutter, 2012). The core personality features associated with psychopathy are callous and unemotional personality traits, which include a lack of empathy or remorse, weak social bonds, an uncaring nature, and shallow emotional responding (Cooke et al., 2005; Frick and White, 2008; Viding and McCrory, 2012). The antisocial behavior tendencies that tend to accompany these traits include poor control of anger, impulsiveness, irresponsibility, and a parasitic orientation toward others (Frick and Ellis, 1999). These factors are generally positively related, such that higher levels of callous and unemotional personality traits predict increased antisocial behavior (Viding et al., 2007; Kahn et al., 2013). The presence of psychopathic traits are particularly strong predictors of aggression that serves an instrumental goal, such as bullying, sexual violence, or assault during the course of a robbery (Blair, 2001; Woodworth and Porter, 2002). Debates persist as to whether the features of psychopathy are best classified using various two-, three-, and four-factor models that have been proposed (Jones et al., 2006; Skeem et al., 2011), and whether criminal or aggressive behavior is an essential part of the psychopathy construct (Hare and Neumann, 2010; Skeem and Cooke, 2010), however, the basic features that compose the construct of psychopathy are generally agreed upon.

Psychopathy is not a clinical diagnosis in the Diagnostic and Statistical Manual (DSM-IV-TR), although features of psychopathy are incorporated into the Axis II diagnosis Antisocial Personality Disorder (Lynam and Vachon, 2012). Various suggestions for updating the DSM 5 to reflect current conceptualizations of psychopathy in adults and children have been proposed (Frick and Moffitt, 2010; Skodol et al., 2011). That said, emerging evidence suggests that psychopathy is not taxonomic in structure. As is the case for traits that comprise other forms of mental illness (Markon et al., 2011), psychopathic traits appear to be continuously distributed in the population and can be most reliably and validly assessed when treated as a continuous rather than a discrete measure (Edens et al., 2006; Guay et al., 2007; Kotov et al., 2011). This is important because it suggests that information about psychopathy can be drawn from both clinically diagnosed samples and community samples (Malterer et al., 2010).

Psychopathy affects both children and adults. Markers of psychopathy may emerge early in childhood (Glenn et al., 2007; Wang et al., 2012), are moderately reliable predictors of adult psychopathy (Lynam et al., 2008), and the core affective features of psychopathy appear to be highly heritable (Larsson et al., 2006). The heritability coefficient of the core callous and unemotional features has been estimated to be at least 0.43 (Larsson et al., 2006) and as high as 0.71 (Viding et al., 2005, 2008). An individual's risk for engaging in antisocial behavior during childhood or adulthood can be increased by any number of life history events, including trauma exposure, low socioeconomic status, or delinquent peer groups (Lynam et al., 2008), but these factors do not seem to precipitate the emergence of psychopathic traits in children (often termed callous-unemotional traits). In fact, callous-unemotional traits may paradoxically serve as a protective factor against parental maltreatment: among children with callous-unemotional traits, there is little correspondence between the quality of parenting that children receive and the severity of their antisocial behavior problems (Wootton et al., 1997). Instead it appears that life stressors that result in heightened stress responding represent a distinct etiological route toward antisocial behavior (Blair, 2001). Among children without high levels of callous-unemotional traits, parental maltreatment is associated with increased antisocial behavior (Wootton et al., 1997). In addition, antisocial behavior in the absence of callous-unemotional traits does not appear to be highly heritable, supporting the role of environmental stressors in leading to antisocial behavior in the absence of callous-unemotional traits (Viding et al., 2005, 2008).

## Psychopathy and fear responding

From the earliest formal clinical descriptions of psychopathy, the construct has been linked to deficient fear responding. Most modern conceptualizations of psychopathy are based on the work of Cleckley (1988), whose compiled observations of institutionalized psychopaths are described in *The Mask of Sanity*. He distinguishes psychopaths from other psychiatric patients as typically free from delusions or irrational thinking, suicidality, or other self-harm tendencies, and, in particular, from anxiety or fear. The second criterion Cleckley specifies for identifying psychopathy is an, “Absence of nervousness or psychoneurotic manifestations,” and he describes the prototypical psychopath as “incapable of anxiety” (p. 340) showing “immunity from … anxiety or worry” (p. 339), and being “free from … nervousness” (p. 339).

Although Cleckley's descriptions of psychopathy reflect a psychodynamic orientation, his observations are consistent with more recent experimental data assessing fear responding in psychopathy. A focus on fear responding emerged from the observation that psychopathic offenders are particularly likely to re-offend, suggesting that the threat of future punishments is not sufficiently motivating for them (Corrado et al., 2004; Hare, 2006). Fear is, in essence, *the state that accompanies the anticipation of an aversive outcome* (i.e., punishment) *and promotes avoidance and escape behaviors* (Stein and Jewett, 1986; Panksepp, 1998; LeDoux, 2000). Fear being the emotion that promotes avoidance of behaviors that result in punishment (LeDoux, 2003), it is ostensibly is the mechanism by which punishing criminal behavior serves to deter it. Early hypotheses proposed that dysfunctional fear responding renders psychopaths less likely to avoid engaging in criminal behaviors that result in punishments like imprisonment, and were supported by laboratory findings that psychopaths are less likely to modulate their behavior in response to anticipated punishments ranging from electrical shock to loss of points in a computer game (Lykken, 1957; Hare, 1966; Newman and Kosson, 1986; Blair et al., 2004).

Abundant psychophysiological research supports the notion that psychopaths' responses to the threat of an aversive outcome are muted. Psychopathy impairs anticipatory skin-conductance responses (Lykken, 1957; Aniskiewicz, 1979; Herpertz et al., 2001; Birbaumer et al., 2005; Rothemund et al., 2012), fear-potentiated startle responses (Patrick et al., 1993; Levenston et al., 2000; Herpertz et al., 2001; Rothemund et al., 2012), and contraction of the corrugator muscle underlying the brows (Herpertz et al., 2001; Rothemund et al., 2012) during threat anticipation. Psychopathy also impairs aversive classical conditioning (Flor et al., 2002) as well as other fear-relevant responses such as the recognition of fear from the face, body, and voice (Marsh and Blair, 2008; Dawel et al., 2012). These differences are particularly evident for psychopathic offenders characterized as “primary” psychopaths who exhibit the core callous and unemotional personality features of the disorder (Lykken, 1957; Aniskiewicz, 1979; Dawel et al., 2012). This is in contrast to “secondary” psychopaths, in whom antisocial behavior may primarily reflect social disadvantage or maltreatment and who may present with increased anxiety (Newman et al., 2005; Kimonis et al., 2012).

Finally, both anecdotal reports and empirical evidence indicate that subjective experiences of fear are reduced in psychopathy. In *Without Conscience* (Hare, 1993), Hare describes an interview with a psychopathic offender who seemingly fails to understand the fundamental nature of fear:

Another psychopath … said that he did not really understand what others meant by “fear.” However, “When I rob a bank,” he said, “I notice that the teller shakes or becomes tongue-tied. One barfed all over the money. She must have been pretty messed up inside, but I don't know why. If someone pointed a gun at me, I guess I'd be afraid but I wouldn't throw up.” When asked to describe how he *would* feel in such a situation, his reply contained no references to body sensations. He said things such as, “I'd give you the money”; “I'd think of ways to get the drop on you”; “I'd try and get my ass out of there.” When asked how he would *feel*, not what he would think or do, he seemed perplexed. Asked if he ever felt his heart pound or his stomach churn, he replied, “Of course! I'm not a robot. I really get pumped up when I have sex or when I get into a fight” (pp. 53–54).

Also supporting reduced subjective experience of fear in psychopathy are the results of a recent study in which adolescents with psychopathic traits and healthy controls underwent an autobiographical recall paradigm adapted from a task developed to measure subjective experiences of emotion across cultures (Scherer and Wallbott, 1994). In the task, participants described recent emotionally evocative events and their subjective responses during these events. This paradigm has the advantage of using a single measure to assess responses to five emotional states. Relative to controls, adolescents with psychopathic traits reported reduced symptoms of sympathetic nervous system activation, such as changes in breathing or muscle tension, during fear-evoking events, even though judges rated the psychopathic adolescents' descriptions of the fear-evoking events as no less inherently frightening than the events reported by controls. In addition, psychopathic adolescents reported that in daily life they experience fear less often and less intensely than did controls (Marsh et al., 2011). Two adolescents with psychopathic traits in this study reported *never* having felt fear, an experience not reported by any of the healthy adolescents.

In keeping with this pattern, many contemporary assessments of psychopathy specifically index items related to reduced anxiety and fearfulness. These measures include the Triarchic Psychopathy Measure, e.g., “I'm afraid of far fewer things than most people” (Patrick, 2010); the Youth Psychopathy Inventory e.g., “What scares others usually doesn't scare me” (Andershed et al., 2002); and the Psychopathic Personality Inventory, e.g., “I can remain calm in situations that would make many other people panic” (Lilienfeld and Andrews, 1996). Researchers who use psychopathy measures that do not explicitly include anxiety and fear-relevant items often supplement the scale with anxiety measures or clinical assessments of anxiety disorders (Sutton et al., 2002; Finger et al., 2008; Malterer et al., 2008; Marsh et al., 2008; Kimonis et al., 2012; Koenigs et al., 2012).

In contrast to fear, other forms of emotional responding in psychopathy appear to be spared. The clearest example is anger, which appears intact and perhaps enhanced in psychopathy. Anger can be defined as the *high arousal state that follows frustration or perceived threat and, behaviorally, is closely linked to aggression against the source of frustration or threat* (Blair, 2012). Elevated anger responding is intrinsic to many descriptions of psychopathy. Both Cleckley and Hare's case studies include numerous descriptions of psychopaths whose misbehavior included frequent temper tantrums and rage-induced aggression. And contemporary measures of psychopathy universally feature items that index frequent, heightened, or undercontrolled displays and experiences of anger. These measures include the youth and adult variants of the Psychopathy Checklist, e.g., “Poor anger control” (Forth et al., 2003); the Antisocial Processes Screening Device, e.g., “Becomes angry when corrected or punished” (Frick and Hare, 2001); the Levenson Self-Report Psychopathy Scale, e.g., “When I get frustrated, I often ‘let off steam’ by blowing my top” (Levenson et al., 1995), and the Psychopathic Personality Inventory, e.g., “From time to time I really ‘blow up’ at other people” (Lilienfeld and Andrews, 1996). That these criteria are positively correlated with the overall construct reinforces the positive relationship between psychopathy and anger experiences.

In psychopathy, anger is most likely to result from goal frustration rather than perceived threat (Blair, 2012), although it should be noted that considerably less empirical research has assessed anger responding in psychopathy compared to fear. That said, three recent studies have found psychopathy to be associated with intact or heightened anger responding both physiologically and subjectively. Hicks and Patrick (2006) evaluated angry responding using a series of self-report scales and found elevated anger responding in psychopathy, with closer associations found between angry responding and the antisocial behavior subscale. In a similar vein, Blackburn and Lee-Evans (2011) found that psychopathic participants anticipated that they would respond with greater anger than non-psychopaths to a variety of anger-inducing scenarios. Lobbestael et al. (2009) performed an anger induction task in individuals with Antisocial Personality Disorder (who varied in psychopathic traits), Borderline Personality Disorder and controls. The induction task entailed recalling a situation in which subjects had experienced a conflict with another person and had felt very angry, after which subjects spent several minutes recalling the details of the event. Results indicated that neither total psychopathy scores nor callous and unemotional personality trait scores among individuals with antisocial personality disorder were predictive of physiological changes during the anger induction task, suggesting an intact anger response. Other studies have found no group differences in responses linked to anger, such as the study assessing subjective experiences of emotion in psychopathic adolescents and controls (Marsh et al., 2011), and the results of two meta-analyses assessing the recognition of anger from the face, body, or voice (Marsh and Blair, 2008; Dawel et al., 2012).

A second emotional state that appears to be intact in psychopathy is positive excitement. This state can be distinguished from happiness, which is more closely associated with goal attainment, as the *state that accompanies the anticipation of an appetitive outcome* (i.e., reward) *and promotes acquisition or achievement of the reward*—a state that is in some ways a mirror image of fear and that has been alternately termed wanting, seeking, or interest (Berridge et al., 2009). The quotation from the incarcerated psychopath above is suggestive of the presence of positive excitement in psychopathy, and is consistent with clinical observations and empirical data that psychopaths are positively motivated by the prospect of reward, particularly near-term reward. Cleckley's criteria include several items that describe unrestrained goal-seeking in the context of money, sexual gratification, and other rewards (Cleckley, 1988). And, as is true for anger, contemporary measures of psychopathy feature items related to the experience of wanting, seeking, and excitement, including the Psychopathy Checklist, e.g., “Stimulation seeking” (Forth et al., 2003); the Youth Psychopathy Inventory, e.g., “If I get the chance to do something fun, I do it no matter what I had been doing before” (Andershed et al., 2002); the Levenson Self-Report Psychopathy Scale, e.g., “My main purpose in life is getting as many goodies as I can” (Levenson et al., 1995), and the Psychopathic Personality Inventory, e.g., “If I were a firefighter, I think I might actually enjoy the excitement of trying to rescue someone from the top floor of a burning building” (Lilienfeld and Andrews, 1996). Empirical behavioral data also exist to suggest that the motivational salience of rewarding stimuli is similar to that of comparison samples (Blair et al., 2004) or perhaps even increased (Scerbo et al., 1990; Bjork et al., 2012). Because positive excitement is not always included on lists of basic emotion it is subject to less focused research than emotions like anger and fear. However, what evidence exists suggests that this state is intact or heightened in psychopathy.

There is very little evidence available that describes other types of emotional reactions in psychopathy, although what evidence exists suggests that disgust responding remains intact, and there is little evidence for consistent impairments in happiness or surprise (Marsh and Blair, 2008; Marsh et al., 2011; Dawel et al., 2012). One emotion for which the present literature is genuinely ambiguous is sadness, with meta-analytic findings generally showing some deficits in recognizing sadness expressions in psychopathy, albeit less consistently and with generally smaller effect sizes than for fear. Very little literature explores sadness responses in psychopathy in other contexts, and results from these studies are equivocal (e.g., Blair et al., 1995; Brook and Kosson, 2013) In general, the neurobiological basis of sadness is not as well understood as that of fear, and further development of the neurocognitive basis of sadness may be required to develop targeted tasks assessing it in psychopaths.

It should be noted that among Cleckley's original criteria is “General poverty in major affective reactions” which is reflected in items measuring shallow affect in contemporary measures such as the PCL variants and APSD (Hare, 1991; Frick and Hare, 2001). However, Cleckley's emphasis is primarily the quality of the anger, excitement, etc. that psychopaths experience—how long-lasting these states are, how consistent, and how “mature” their expression. Thus, whereas psychopaths may display outward signs of rage and become “vexed,” “peevish,” or “resentful,” Cleckley proposes that they do not experience “mature, wholehearted anger” (Cleckley, 1988, p. 348). The lability or consistency of affective reactions in psychopathy may be an important feature of the disorder. However, it remains the case that among basic emotions, only in the case of fear does strong, consistent empirical evidence support the existence of deficits in psychopathy.

## Are emotions discrete natural kinds or constructed using dimensions of core affect?

These patterns of observed emotional responding in psychopathy may help to explicate a central ongoing question about emotion, namely: can emotions be better described as *qualitatively* distinct, for example, as discrete “basic emotions” or “natural kinds” (Ekman et al., 1983; Izard, 1992; Panksepp, 2005) or as *quantitatively* distinct, for example, as points along a circumplex defined by dimensions like arousal and valence (Russell and Barrett, 1999; Barrett and Wager, 2006)? Recent years have seen a protracted debate in the literature about how to most accurately capture the nature of emotion (Barrett et al., 2007; Izard, 2007; Panksepp, 2007; Tracy and Randles, 2011), with proposed models of emotion including not only basic emotion and dimensional models, but also those that focus upon goal-relevant appraisals of emotional stimuli (Moors et al., 2013), emotions as coping responses (Roseman, 2013), and emotions as survival circuits (LeDoux, 2012). An extended conversation about the strengths and weaknesses of these various views will not be reviewed in full here, rather, the focus will be on the basic consideration of whether different emotions (e.g., fear, anger) are best viewed as qualitatively or quantitatively distinct.

Models that posit emotions to be qualitatively distinct, such as “basic emotion” models, holds that a limited number of emotions like fear, anger, and positive excitement emerge from dissociable neurophysiological processes (Ekman et al., 1983; Izard, 1992; Panksepp, 2005; Lench et al., 2011). These neurophysiological processes are generally linked to activity in the evolutionarily ancient subcortical structures of the midbrain, striatum, and limbic system most commonly linked to emotion (Panksepp, 2005; Vytal and Hamann, 2010). So, for example, the generation of positive excitement is linked to activation in a striatal circuit centered on dopaminergic neurons in the nucleus accumbens (Ikemoto and Panksepp, 1999), whereas the generation of fear is associated with activity in a circuit involving the periaqueductal gray, anterior and medial hypothalamus, and amygdala (LeDoux, 2000). In this view, finer gradations of experience result when basic emotions are modulated or elaborated by higher-level cognitive processes controlled by the cerebral cortex, but the emergence of qualitatively distinct emotions is not dependent on these cortically-controlled processes (Panksepp, 2005).

Models that posit emotions to be quantitatively distinct hold that emotions like fear, anger, and happiness are best described as points on one or more core dimensions. Core dimensions typically proposed to distinguish among emotions are physiological arousal or activation (low—high) and valence (bad—good) (Bradley et al., 2001). [Some have proposed a withdrawal—approach dimension as a substitute or supplement to the valence axis (Wager et al., 2003; Christie and Friedman, 2004; van Honk and Schutter, 2006)]. Arranged orthogonally, these dimensions form a circumplex upon which emotions can be plotted and quantitatively compared (Barrett and Russell, 1999; Russell and Barrett, 1999; Colibazzi et al., 2010). Positive excitement is plotted as high in arousal and positive in valence, and sadness is low in arousal and negative in valence. Fear is typically plotted as high arousal and strongly negative, as is anger (Russell and Barrett, 1999). Further distinctions among emotions are thought to reflect differences in cognitive construals of the events surrounding the basic changes in arousal and valence. Thus, whether an individual experiences anger or fear (which are similar in terms of arousal or valence) may be shaped by interpretations of neurophysiological changes in valence and arousal in light of the eliciting stimulus and the individual's idiosyncratic stores of semantic knowledge, memories, and behavioral responses that shape the subjectively experienced state (Russell, 2003). Under this view, distinctions among experienced emotional states are highly dependent on these cognitively complex processes, which are subserved by a distributed network of regions of the cerebral cortex (Lindquist et al., 2012).

These models generate distinct predictions to the question of whether a disorder or lesion could result in a single emotion being disabled without affecting the experience of other emotions. The discrete emotions view would argue that a disorder or lesion that resulted in dysfunction in the specific structures subserving a particular emotion could affect the experience of one emotion while leaving others intact. In contrast, the dimensional view would require either that other emotions that are dimensionally similar to the affected emotion also be affected, or that deficits in a particular emotion would reflect dysfunction in cortically-driven higher-level cognitive processes.

The case of psychopathy lends clear support to notion that fear is qualitatively distinct from other emotions. In psychopathy, the bulk of the clinical and empirical evidence points toward the conclusion that fear responding is uniquely disabled, with other high-arousal (positive excitement, anger) and negatively valenced (anger, disgust) emotions remaining intact. The dimensional view cannot easily explain why in psychopaths the high arousal, negatively valenced state of anger is easily (perhaps too easily) generated, whereas the high arousal, negatively valenced state of fear is not. The problem cannot lie in a failure to fully engage neurocognitive systems underlying either the arousal or valence dimension, because psychopaths experience other high-arousal emotions (positive excitement) as well as other negatively valenced emotions (disgust). It also cannot result from some difficulty arising at the interaction of these axes, because anger and fear are highly similar in terms of both dimensions. Models that substitute a withdrawal—approach axis for a negative—positive axis are no more successful; the two most strongly withdrawal-linked emotions are disgust and fear, and there is no evidence for disgust-based impairments in psychopathy.

Can cognitive construals of emotion explain the patterns observed in psychopathy? Perhaps, one could argue, psychopaths under threat are less likely to construe their negative, high-arousal state as fear and more likely to construe it as anger compared to non-psychopaths. So, for example, the psychopath whose interview is transcribed above might interpret a pounding heart and churning stomach as the angry response that accompanies a tendency to respond aggressively. Another person might interpret the same body symptoms as the fear that accompanies a tendency to escape or submit. Theoretically, this explanation could explain both the deficits in fear and a concomitant increase in anger in this population. One could argue that, particularly for studies that focus on subjective reports of emotion, group differences in construal underlie the tendency of psychopaths to underreport experiencing fear and overreport experiencing anger.

This argument suffers two shortcomings. First, it is inconsistent with psychophysiological findings of overall reduced arousal during threat anticipation in psychopathy. As described above, there are two major categories of anger elicitors: perceived threat and goal frustration (Blair, 2012). The construal argument would require that psychopaths experience arousal in response to threat, but interpret this arousal as anger rather than fear. But the evidence is clear that psychopaths (particularly primary psychopaths) are no more likely than average to experience physiological arousal under conditions of threat (Blackburn and Lee-Evans, 2011)—and in fact, as described previously, show reduced physiological responses, including reduced skin conductance, potentiated startle, and corrugator muscle activity. This suggests that threat anticipation results in neither fear nor anger in this population. Psychopaths are, however, more likely than average to experience anger is in response to frustration (Blair, 2012). Thus, rather than being chronically likely to construe any high arousal state as anger, psychopaths appear more likely to experience anger primarily in response to frustrated attempts to achieve a reward. That both frustration-based anger and positive excitement (the state that reflects the anticipation of reward) are normal or elevated in psychopathy is consistent with the notion that in psychopaths the systems that govern anticipation of reward are functional and perhaps even overactive while the systems that govern threat anticipation are dysfunctional. A further concern is that the construal explanation of emotion leaves unclear *why* psychopathy might engender such a dramatic shift in emotional experience. Such a phenomenon is particularly difficult to explain in light of the high heritability coefficient found for psychopathy. Cognitive construals of emotional states are thought to reflect the individual's autobiographical memories and semantic knowledge of emotion prototypes, phenomena that are necessarily a result of learning, rendering it unlikely that the tendency to construe one's emotional response to an event as fear versus anger would itself be heritable.

The pattern of reduced fear responding to anticipated threat observed in psychopathy, then, is more consistent with the view that states like anger and fear reflect biologically coherent and qualitatively distinct responses to particular eliciting stimuli. Dimensions like valence and arousal are useful means of quantitatively describing differences among subjective feeling states like fear, anger, and positive excitement, but may not accurately reflect the neurobiological origins of those states.

## What are the brain structures involved in generating specific emotions like fear?

If psychopathy is associated with specific deficits in fear responding, this not only supports the idea that emotions are qualitatively distinct, it supports the corollary that specific neurophysiological processes that support the fear response are also affected. A key feature of models of discrete emotions is that distinct emotions have dissociable neurophysiological correlates (Vytal and Hamann, 2010). Ekman (1999) has argued:

The distinctive features of each emotion, including the changes not just in expression but in memories, imagery, expectations, and other cognitive activities, could not occur without central nervous system organization and direction. There must be unique physiological [CNS] patterns for each emotion (p. 50).

Limited evidence exists to suggest specific patterns of peripheral nervous system activity that accompany discrete emotions (Ekman et al., 1983; Christie and Friedman, 2004), however, assuming that the origins of basic emotions are in the central nervous system, most research in this vein has focused on the central origins of emotions, specifically, the structures or networks of brain structures in which activity supports the emergence of particular emotions (Panksepp, 2007; Vytal and Hamann, 2010; Lindquist et al., 2012).

The availability of non-human animal analogues has made fear one of the best-studied emotions on a neuroanatomical level. On the whole, the empirical data support the idea that the amygdala, along with its efferent projections, is an essential structure for the generation of conditioned fear responses, which account for the majority of experienced fear (Davis, 1992, 1997). [Unconditioned fear in response to specific events like carbon dioxide-induced air hunger may rely on distinct neural pathways (Johnson et al., 2011; Feinstein et al., 2013)]. Extensive early evidence demonstrated that the amygdala plays a crucial role in the creation of conditioned fear in rodents. For example, lesions to the amygdala prevent rats from developing a conditioned fear response, like freezing in response to a stimulus that predicts shock (Blanchard and Blanchard, 1972). Later studies clarified the roles of the various subnuclei of the amygdala, demonstrating that the lateral nucleus is primarily involved in the acquisition of the fear response whereas the central nucleus is involved in both the acquisition and the expression of conditioned fear responses (Davis, 1992; Wilensky et al., 2006). The amygdala's many efferent projections coordinate autonomic and behavioral responses to fear eliciting stimuli. Projections from the central nucleus of the amygdala to the lateral hypothalamus are involved in activating autonomic sympathetic nervous system responses, and projections to the ventrolateral periaqueductal gray direct the expression of behavior responses, such as defensive freezing (Davis, 1992; LeDoux, 2012). The amygdala's central role in coordinated fear responding can be demonstrated by electrical stimulation studies showing that complex patterns of behavioral and autonomic changes associated with fear responses result from stimulation of the relevant regions of the amygdala (Davis, 1992). Heavy reliance on animal models is justified in the study of fear responding and the amygdala given how strongly conserved the amygdala nuclei involved in responding to conditioned threats are across species ranging from reptiles to birds to rodents to primates (LeDoux, 2012).

Ethical and pragmatic considerations prevent experimental paradigms employing electrical stimulation or ablation of the amygdala from being undertaken in human subjects. However, the advent of neuroimaging technologies have enabled considerable assessments of subcortical responses to a variety of emotional stimuli, enough to provide a basis for seven meta-analyses that have been conducted to assess patterns of brain activation in response to specific emotions (Phan et al., 2002; Murphy et al., 2003; Kober et al., 2008; Sergerie et al., 2008; Fusar-Poli et al., 2009; Vytal and Hamann, 2010; Lindquist et al., 2012). The findings from four of these meta-analyses support the role of the amygdala in human fear responding. Phan and colleagues reviewed 55 PET and fMRI studies (including 13 that assessed fear responding) and found that fear specifically activated the amygdala relative to other emotions (Phan et al., 2002). Sixty percent of studies assessing fear responses observed an increased amygdala response whereas fewer than 25% of other emotional tasks resulted in amygdala activation increases. Murphy and colleagues reviewed 106 PET and fMRI studies (Murphy et al., 2003) and again observed the most consistent amygdala responses during the induction or perception of fear relative to other emotions, interpreting their data as consistent with amygdala specialization for fear. In neither meta-analysis was any other structure observed to be consistently and selectively activated during fear paradigms. Fusar-Poli and colleagues included only fMRI studies assessing responses to emotional faces, but again found heightened amygdala responses to fearful faces relative to other emotional faces (Fusar-Poli et al., 2009). Finally, Vytal and Hamann (2010) employed a more sensitive meta-analytic method, activation likelihood estimation (ALE), to analyze the results of 83 PET and fMRI studies of emotion (including 37 that assessed fear responding) and again found strong support that the amygdala is preferentially active during fear paradigms, and this activation in this region differentiated fear from happiness, sadness, and disgust.

Three recent meta-analyses did not yield findings that fear is preferentially associated with amygdala activation. Two were conducted by Feldman-Barrett and colleagues (Kober et al., 2008; Lindquist et al., 2012). In the more recent analysis, Lindquist and colleagues analyzed 91 fMRI and PET studies of emotion, including 42 assessing fear (Lindquist et al., 2012). The authors observed that, bilaterally, the amygdala was the most active brain region during fear perception paradigms (although not significantly more active during fear than other emotions), but that the amygdala was not preferentially active during fear experience paradigms. The selection of studies in this meta-analysis may account in part for the differential findings. For example, of the nine fear-experience studies included in this analysis, six were conducted by a group that uses primarily IAPS pictures (Lang et al., 1999) and similar images to elicit disgust and fear (e.g., Stark et al., 2003; Schienle et al., 2005). These studies may be problematic because many of the “fear” images they use explicitly depict strong non-fear emotional cues (human or animal anger expressions) or depict events like a car accident or lava covering a road that are unpleasant but not obviously frightening. These meta-analyses also omitted pain anticipation and mood induction tasks included in other meta-analyses that are more directly relevant to fear experience (Murphy et al., 2003; Vytal and Hamann, 2010). The third meta-analysis (Sergerie et al., 2008) also excluded pain anticipation and mood induction tasks, in addition to employing a distinct analytical approach, whereby the authors compiled the statistical effect sizes of all studies of emotion (148 in total) that reported any activation in the amygdala and its surrounding regions. This approach yielded results showing amygdala activation that was stronger in response to positive emotional stimuli than to any negative emotional stimuli. Clearly, the conclusions drawn from the various meta-analyses are divergent enough to leave questions remaining as to whether the amygdala is in fact specifically implicated in fear responding.

Can the study of psychopathy clarify the role of the amygdala in fear experience? Perhaps, given the prominence of dysfunctional fear responding in psychopathy, empirical support that amygdala dysfunction underlies aberrant fear responding in psychopathic participants would support the amygdala's role in fear. And indeed, early hypotheses about the brain basis of psychopathy focused on potential amygdala dysfunction (Patrick, 1994; Blair et al., 2001). More recently, the results of both functional and structural neuroimaging studies support these hypotheses. Several studies have observed that psychopathy is associated with reduced amygdala activation during the viewing of fearful emotional facial expressions but not other expressions like anger, a pattern that is independent of attentional processes (Marsh et al., 2008; Dolan and Fullam, 2009; Jones et al., 2009; White et al., 2012). A recent study also found that psychopathy assessed in a community sample was also associated with a failure to exhibit amygdala activation to fear-evoking statements (Marsh and Cardinale, 2012b). Again, no group differences were observed in this task when other emotionally evocative statements were presented. (In addition, no main effect of fear stimuli was observed in the amygdala across groups. This suggests that amygdala responses to fear may fail to emerge in neuroimaging studies when the sample contains an unusual proportion of high psychopathy scorers.) Finally, a fear-conditioning paradigm found that psychopaths' failure to exhibit skin conductance responses during the task was accompanied by reduced activation in the amygdala and functionally connected regions of the cortex, such as orbitofrontal cortex and insula (Birbaumer et al., 2005).

These patterns of dysfunction may stem from structural abnormalities in the amygdala, which have also been observed in psychopathy. Structural abnormalities across multiple nuclei in the amygdala have been observed in psychopathy (Yang et al., 2009, 2010; Ermer et al., 2012). Yang and colleagues observed not only significant bilateral volume reductions in the amygdalae of adult psychopaths relative to controls controls, but also surface deformations in the vicinity of the amygdala's basolateral, lateral, cortical, and central nuclei. A later study indicated that these deformities are more significant in “unsuccessful” psychopaths, or those who have been prosecuted for their criminal acts (Yang et al., 2010). Ermer and colleagues identified gray matter reductions in adult psychopaths' amygdalae, in addition to other paralimbic regions such as parahippocampal gyrus (Ermer et al., 2012). It should be noted that how specific nuclei of the amygdala are involved in psychopathy is not yet clear, in part due to insufficient spatial resolution of functional imaging scan. Various hypotheses have been proposed regarding the role of discrete nuclei in psychopathic symptoms (Blair, 2005a; Moul et al., 2012).

On the whole, the results of these studies directly link amygdala dysfunction to observed deficits in fear responding in psychopathy.

But perhaps the most compelling evidence that amygdala dysfunction underlies fear deficits in psychopathy emerges from the results of paradigms testing fear responding in psychopaths and individuals with lesions to the amygdala. As previously described, psychopathy has been found to impair anticipatory skin-conductance responses (Lykken, 1957; Aniskiewicz, 1979; Herpertz et al., 2001; Birbaumer et al., 2005; Rothemund et al., 2012), fear-potentiated startle responses (Levenston et al., 2000; Herpertz et al., 2001; Rothemund et al., 2012), aversive classical conditioning (Flor et al., 2002), subjective experiences of fear (Marsh et al., 2011) and the recognition of fear from the face, body and voice (Marsh and Blair, 2008; Dawel et al., 2012). Striking parallels to these deficits can be found in studies of individuals with amygdala damage. In these individuals, comparable impairments in each of these fear paradigms have also been observed (Table 1).

**Table 1:** **Comparison of deficits observed in samples with psychopathy and amygdala lesions**.

	**Psychopathy**	**Amygdala lesions**
Potentiated startle	Levenston et al., 2000; Herpertz et al., 2001	Angrilli et al., 1996; Buchanan et al., 2004
Anticipatory SCR	Hare, 1982; Ogloff and Wong, 1990; Rothemund et al., 2012	Bechara et al., 1995
Aversive conditioning	Lykken, 1957; Flor et al., 2002	LaBar et al., 1995; Bechara et al., 1999
Facial fear recognition	Blair et al., 2004; Marsh and Blair, 2008	Adolphs et al., 1994, 1999
Vocal fear recognition	Blair et al., 2002, 2005	Scott et al., 1997; Sprengelmeyer et al., 1999
Postural fear recognition	Munoz, 2009	Sprengelmeyer et al., 1999
Reduced subjective fear	Marsh et al., 2011	Masaoka et al., 2003; Feinstein et al., 2011

Because amygdala dysfunction has been observed in psychopathy during several of these tasks, and because amygdala lesions impair performance in all of them, these patterns generate a compelling case for the role of the amygdala specifically in fear responding. Consistent with this, researchers studying one patient with bilateral amygdala damage (SM) clarify that she has not only striking deficits in fear responding, but these deficits are limited to fear responding:

SM's reaction to fear-inducing stimuli was not characterized by a loss of responsiveness, but rather manifested as a heightened arousal and interest in the face of a near-complete lack of avoidance and caution … Our findings suggest that the amygdala's role in the induction and experience of emotion is specific to fear. To say that SM is emotionless or unable to feel emotion is simply false. Her emotional deficit is primarily circumscribed to the behaviors and experiences that characterize a state of fear (Feinstein et al., 2011).

The clear correspondence between patterns of fear dysfunction observed in psychopathy and following amygdala lesions, in the absence of other clear emotional deficits, provides strong support for the specific involvement of the amygdala in fear. Dysfunction in the amygdala, whether via acquired lesion or developmental psychopathology, impairs fear-related processes while leaving other forms of emotion, such as anger, positive excitement, and disgust, largely intact. In answer to our second question, then, research in psychopathy suggests that the amygdala—or, more likely, specific populations of neurons within the amygdala (LeDoux, 2012)—plays a critical role in generating fear but does not appear to be critical for other emotions like positive excitement and anger.

## How do our own experiences of emotion pertain to our perceptions of and responses to others' emotion?

The findings reviewed thus far suggest answers to a third question of ongoing interest in psychology and neuroscience: how do our emotional experiences affect our responses to and perceptions of others' emotions?

As we have seen, the evidence is clear that psychopathy is associated with deficits in the experience of fear but not other emotions. Psychopathic individuals show reduced physiological responding during anticipation of an aversive event, are less apt to adapt their behavior in response to punishment, and report reduced subjective fear. In some psychopaths the experience of fear may be essentially absent but, in keeping with the idea that psychopathy is a continuum rather than a taxon, fear is likely muted to varying degrees rather than absent in most individuals with psychopathic traits. Finally, psychopathy impairs the recognition of others' fear. Three meta-analyses have now demonstrated that psychopathy impairs recognition of fearful facial expressions in the face, body, and voice (Marsh and Blair, 2008; Wilson et al., 2011; Dawel et al., 2012), a pattern that is particularly closely associated with the central affective deficits of psychopathy. Marsh and Blair (2008) found that responses to fear are impaired to a significantly greater degree than any other emotion, and Dawel et al. (2012) found that the core affective features of psychopathy impaired the recognition of fear but not other emotions. In addition, psychopathy impairs the ability to identify the circumstances under which others would experience fear, such as in response to threats of harm (Marsh and Cardinale, 2012a). The parallels between psychopathic deficits in emotional experience and emotion recognition are striking. The emotion that psychopaths appear not to feel strongly—fear—is the same emotion that they have the most difficulty recognizing in others. Associations between the experience and recognition of emotion have previously been observed for a number of emotions, including fear (Buchanan et al., 2010). These data suggest the possibility of a basic empathic failure in psychopaths—they have great difficulty understanding an emotion in others that they themselves do not feel (or at least, do not feel strongly). This breakdown appears to occur in primarily for fear, rendering others' expressions of fear essentially meaningless in individuals with psychopathic traits.

These patterns are consistent with the theory that we recognize others' emotions through a low-level empathic simulation process, exploiting our own experiences of an affective state to understand others' experiences (Goldman and Sripada, 2005). Empathic simulation has become a favored explanation among researchers studying empathy for pain, boosted by a voluminous literature that the perception or inference of others' pain results in increased activation in the same brain structures involved in processing affective and motivational features of felt pain (Lamm et al., 2011). It is now widely agreed that the experience of empathy for pain emerges from shared representations for personal and vicarious experiences of affective states (Bernhardt and Singer, 2012).

The neurobiological evidence that empathy for fear also results from shared neural representations is equally compelling: both experienced fear and perceived fear result in specific activation in the amygdala, a structure that, when damaged or dysfunctional (as in the case of psychopathy), leads to impairments in both felt fear and the ability to recognize when others are experiencing fear. And yet an extremely similar pattern of data to support amygdala-based shared representations of fear has been interpreted differently from evidence supporting shared insula and anterior cingulate cortex-based representations for pain.

Why might this be? For one, the functions of the amygdala were first articulated in animal models, with a historical emphasis on stimulus-reinforcement learning rather than social functions and subjective experiences. This emphasis may have resulted in early observations of amygdala activity in response to fear expressions being interpreted as indicating that fear expressions signal threat, akin to the CS+ in a conditioning trial (Breiter et al., 1996; Morris et al., 1996). However, there is little empirical data to support the idea that fear expressions are interpreted as primarily threatening. Indeed, fearful facial expressions have been shown to be more strongly appetitive than aversive (Marsh et al., 2005b), and to resemble the morphological appearance of an infantile face (Marsh et al., 2005a) consistent with the idea that others' fear elicits empathic concern. The assumption that fearful expressions signify threat because they elicit amygdala activation may be a case of erroneous reverse inference—an inference regarding the psychological significance of a stimulus on the basis of neural responses to it (Poldrack, 2008).

Alternate hypotheses exist as well, such as that amygdala responses to fearful expressions reflect the amygdala's role in directing attention to the eyes of these expressions, which is critical to correctly identifying these stimuli (Dadds et al., 2006; Han et al., 2012). This theory is supported by findings that instructing both patients with amygdala lesions and children with psychopathic traits to attend to the eyes of faces reduces fear recognition deficits (Adolphs et al., 2005; Dadds et al., 2006). But this theory is less clearly able to accommodate the facts that psychopathy also impairs pre-attentive recognition of fearful faces (Sylvers et al., 2011), that both amygdala lesions and psychopathy impair recognition of vocalized fear, auditory stimuli for which the relevance of attention directed to salient features is unclear (Scott et al., 1997; Blair et al., 2002), and that psychopathy impairs the recognition of written statements that evoke fear (Marsh and Cardinale, 2012a). No low-level features of fear-evoking statements distinguish them from any other emotionally evocative statement, so there is no obvious mechanism by which the redirection of attention would be relevant to identifying these stimuli. I suggest that the total available evidence can be more parsimoniously interpreted under the hypothesis that amygdala is essential to generating an internal representation of fear, and that amygdala dysfunction in psychopathy impairs this process, thereby impairing identification of others' fear across contexts (Marsh and Cardinale, 2012b). This theory has the benefit of being consistent with the vast and consistent literature on empathy for pain.

That low-level emotional processes may impair empathy for fear in psychopathy may be particularly germane to an understanding of empathic processes more generally. “Empathy” is a term plagued by multiple overlapping definitions that include low-level emotional contagion, cognitive perspective-taking, and empathic concern (de Waal, 2009). The form of empathy most notoriously impaired in psychopathy is empathic concern, sometimes called sympathy, the inverse of which is callousness (Hare, 1991; Blair, 1995). By contrast, the evidence is clear that cognitive empathy, or perspective-taking, is not impaired in psychopathy (Blair, 2008; Jones et al., 2010; Schwenck et al., 2012). But emotional contagion, defined as simple affectedness by another's emotional state (de Waal, 2009), is clearly affected, at least in response to others' fear. The accumulated literature on psychopathy thereby suggests the possibility of critical links among emotional contagion in response to others' fear, recognition of others' fear, and empathic concern (Nichols, 2001). It also reinforces the importance of resisting the temptation to conflate the various forms of empathy, which may rely on distinct neurobiological processes.

From a societal perspective, understanding empathic deficits for others' fear may be the most important question of all that the study of psychopathy helps to answer. Although amygdala lesion cases can illuminate the amygdala's role in fear, because these lesions usually occur in late adolescence or adulthood, their effects on the development of other brain regions and behavior is more limited. This may be why amygdala lesions in adulthood are not associated with heightened aggression, whereas the case of psychopathy suggests a strong relationship between developmental deficits in fear and aggression. Fear plays an important role in preventing or ending aggression during social encounters (Blair, 1995, 2005b), and fearful emotional facial expressions elicit empathic concern and the desire to help from people who perceive them, even subliminally (Marsh and Ambady, 2007). The rationale for much research on psychopathy is that individuals with this disorder are responsible for a disproportionate amount of suffering, as they engage in a variety of antisocial, criminal, and violent behaviors that cause others distress and fear (Hare, 1993; Rutter, 2012). There is limited evidence that failure to exhibit empathic responses to others' pain is related to lower self-reported empathic concern or aggressive or antisocial behavior (Singer et al., 2004, 2006). In contrast, the evidence linking the failure to exhibit empathic responses to others' fear, both on a neural and a behavior level, is abundant. Psychopaths, in whom the failure to recognize others' fear or to generate empathic activation in the amygdala and autonomic nervous system is a hallmark feature, exhibit profound impairments in empathic concern for others and notoriously commit antisocial acts. Thus, as important as the study of psychopathy is for answering fundamental psychological and neuroscientific questions about the nature of emotion and empathy, an improved understanding of emotion and empathy as they pertain to psychopathy may be critical to developing improved means of ameliorating psychopaths' harmful effects on others.

## Conclusions

The study of psychopathy has generated information relevant to addressing three questions of central importance to emotion and affective neuroscience. Evidence collected from psychopathic populations supports the conclusion that fear is qualitatively distinct from other emotions and arises from discrete neurobiological processes, rather than the conclusion that emotions like fear and anger reflect quantitative variations in core dimensions like arousal and valence. Recent neurocognitive and neuroimaging evidence also supports the specific role of the amygdala in generating a fear response over the view that the amygdala plays a domain-general role equally relevant to the generation of multiple emotions. And finally, psychopaths' parallel deficits in experiencing fear and recognizing fear in others lend support to the notion that empathy for affective states results from shared representations for personal and vicarious experiences of fear, consistent with simulation-based theories of empathy. These conclusions may prove useful not only in furthering the neuroscientific studies of emotion, but in developing a better understanding of the fundamental nature of psychopathy, empathy and aggression.

### Conflict of interest statement

The author declares that the research was conducted in the absence of any commercial or financial relationships that could be construed as a potential conflict of interest.
